# Overexpression of the soybean transcription factor GmDof4 significantly enhances the lipid content of *Chlorella ellipsoidea*

**DOI:** 10.1186/s13068-014-0128-4

**Published:** 2014-09-04

**Authors:** Jianhui Zhang, Qiang Hao, Lili Bai, Jin Xu, Weibo Yin, Liying Song, Ling Xu, Xuejie Guo, Chengming Fan, Yuhong Chen, Jue Ruan, Shanting Hao, Yuanguang Li, Richard R-C Wang, Zanmin Hu

**Affiliations:** Institute of Genetics and Developmental Biology, Chinese Academy of Sciences, Datun Road, Chaoyang District, Beijing, 100101 China; College of Life Sciences, University of Chinese Academy of Sciences, No.19A Yuquan Road, Beijing, 100049 China; Beijing Institute of Genomics, Chinese Academy of Sciences, Beichen West Road #1, Beijing, 100029 China; State Key Laboratory of Bioreactor Engineering, East China University of Science and Technology, Meilong Road #130, Shanghai, 200237 China; USDA-ARS, FRRL, Utah State University, 695 N. 1100 E., Logan, UT 84322-6300 USA

**Keywords:** Microalgae, *Chlorella ellipsoidea*, Transcription factor, Lipid accumulation, RNA-seq, Acetyl-coenzyme A carboxylase

## Abstract

**Background:**

The lipid content of microalgae is regarded as an important indicator for biodiesel. Many attempts have been made to increase the lipid content of microalgae through biochemical and genetic engineering. Significant lipid accumulation in microalgae has been achieved using biochemical engineering, such as nitrogen starvation, but the cell growth was severely limited. However, enrichment of lipid content in microalgae by genetic engineering is anticipated. In this study, GmDof4 from soybean (*Glycine max*), a transcription factor affecting the lipid content in *Arabidopsis*, was transferred into *Chlorella ellipsoidea*. We then investigated the molecular mechanism underlying the enhancement of the lipid content of transformed *C. ellipsoidea*.

**Results:**

We constructed a plant expression vector, pGmDof4, and transformed *GmDof4* into *C. ellipsoidea* by electroporation. The resulting expression of *GmDof4* significantly enhanced the lipid content by 46.4 to 52.9%, but did not affect the growth rate of the host cells under mixotrophic culture conditions. Transcriptome profiles indicated that 1,076 transcripts were differentially regulated: of these, 754 genes were significantly upregulated and 322 genes were significantly downregulated in the transgenic strains under mixotrophic culture conditions. There are 22 significantly regulated genes (|log_2_ ratio| >1) involved in lipid and fatty acid metabolism. Quantitative real-time PCR and an enzyme activity assay revealed that GmDof4 significantly up-regulated the gene expression and enzyme activity of acetyl-coenzyme A carboxylase, a key enzyme for fatty acid synthesis, in transgenic *C. ellipsoidea* cells.

**Conclusions:**

The hetero-expression of a transcription factor *GmDof4* gene from soybean can significantly increase the lipid content but not affect the growth rate of *C. ellipsoidea* under mixotrophic culture conditions. The increase in lipid content could be attributed to the large number of genes with regulated expression. In particular, the acetyl-coenzyme A carboxylase gene expression and enzyme activity were significantly upregulated in the transgenic cells. Our research provides a new way to increase the lipid content of microalgae by introducing a specific transcription factor to microalgae strains that can be used for the biofuel and food industries.

**Electronic supplementary material:**

The online version of this article (doi:10.1186/s13068-014-0128-4) contains supplementary material, which is available to authorized users.

## Background

Biodiesel is a renewable and environmentally friendly succedaneum for fossil fuels [1]. Many plants have the potential to be used as resources to produce biodiesel; these plants include algae, oilseed rape, soy, and jatropha, of which microalgae have been regarded for decades as the having the highest potential because they can be grown in waste- or seawater [2,3]. Several species of microalgae have higher biomass production rates than those of terrestrial plants [4]. The energy-rich compounds that microalgae produce, such as triacylglycerol (TAG), can be utilized for biodiesel. Some species of microalgae contain a high oil content; these species include *Botryococcus braunii* with lipid contents of 57 to 64%, *Schizochytrium sp.* with lipid contents of 50 to 77%, and *Neochloris oleoabundans* with lipid contents of 35 to 65%. However, those species grow slowly and have low rates of oil production [5]. In contrast, other species (for instance, *Chlamydomonas reinhardtii*, *Chlorella pyrenoidosa* and *Navicula pelliculosa*) grow rapidly but with low lipid content (<15%) [6,7]. The desirable algal strains for lipid production should have the best combination of biomass productivity and lipid content, which are often inversely related. Therefore, increasing the lipid content of microalgae with a high growth rate is essential for the production of biodiesel that is synthesized from extracted glycerolipid.

*Chlorella ellipsoidea*, a unicellular eukaryotic green alga, can be cultured easily under either autotrophic or heterotrophic conditions [8]. In particular, it can be cultivated in industrial wastewater using CO_2_ that is produced from coal-fired power plants [9]. Compared with other microalgae with a high lipid content, *C. ellipsoidea* has a higher cell growth rate but a slightly lower lipid content [10,11]. The average doubling time of *C. ellipsoidea* on basal medium with glucose is less than 20 hours with a final lipid content of 15% [12,13]. Therefore, increasing the lipid content of *C. ellipsoidea* while maintaining its cell growth rate could make it a desirable resource for producing biodiesel.

To date, genetic engineering, is more promising for increasing the lipid content of microalgae than controlling the nutritional or culture conditions; the latter has been used to increase the lipid content in several microalgae, but the cell growth was severely limited [14,15]. Many encouraging efforts have been reported for the genetic improvement of the lipid content in microalgae. Trentacoste *et al*. successfully increased microalgal lipid accumulation without compromising the growth in *Thalassiosira pseudonana* by a knockdown of a multifunctional lipase/phospholipase/acyltransferase [16]. Niu *et al*. found that the overexpression of an acyl-Coenzyme A:diacylglycerol acyltransferase gene in *Phaeodactylum tricornutum* can increase lipid biosynthesis [17]. In addition to genes that are directly related to lipid metabolism, many valuable transcription factors (TFs) that are involved in the regulation of lipid synthesis in higher plants have been identified in recent years [18]. One excellent example is Dof (DNA binding with one finger) protein, which contains a single C_2_C_2_-type zinc-finger-like motif that specifically recognizes an (A/T) AAAG sequence [19]. Dof plays many crucial roles in regulating many biological processes in plants, including the defense gene expression, seed germination, phytochrome signaling, and photoperiodic control of flowering in *Arabidopsis* [20], as well as the synthesis of seed storage proteins in *Zea mays* [21] and the photosynthesis and sucrose transport in *Triticum aestivum* [22]. In particular, *GmDof4*, which was found in *Glycine max*, was involved in lipid synthesis by activating the acetyl-coenzyme A carboxylase (ACCase) gene via direct binding to the *cis*-DNA elements in its promoter region. The total fatty acid and lipid content in the seeds was significantly increased in *GmDof4*-transformed *Arabidopsis* [23].

The aim of this study is to investigate the feasibility and the mechanism for improving the lipid content of *C. ellipsoidea* by the overexpression of *GmDof4*. Our results indicated that the lipid content of transgenic *C. ellipsoidea* cells was increased by 46.4 to 52.9% under mixotrophic culture conditions, but the contribution of different fatty acids and the growth rate of cells were not significantly affected. Illumina-based RNA-seq results indicated that GmDof4 significantly regulated 1,076 genes of *C. ellipsoidea*, and 22 of these genes were lipid or fatty acid metabolism genes. Real-time PCR analysis and an enzyme activity assay confirmed that the expression and enzyme activity of ACCase were significantly upregulated in transgenic *C. ellipsoidea* cells. These findings proved that GmDof4 was able to activate the expression of ACCase genes specifically. Our results also provided a new route for engineering microalgae to increase the lipid content and shed light on the mechanism of lipid accumulation in microalgae regulated by Dof from higher plant.

## Results

### Identification of transgenic cells

**Figure 1:**
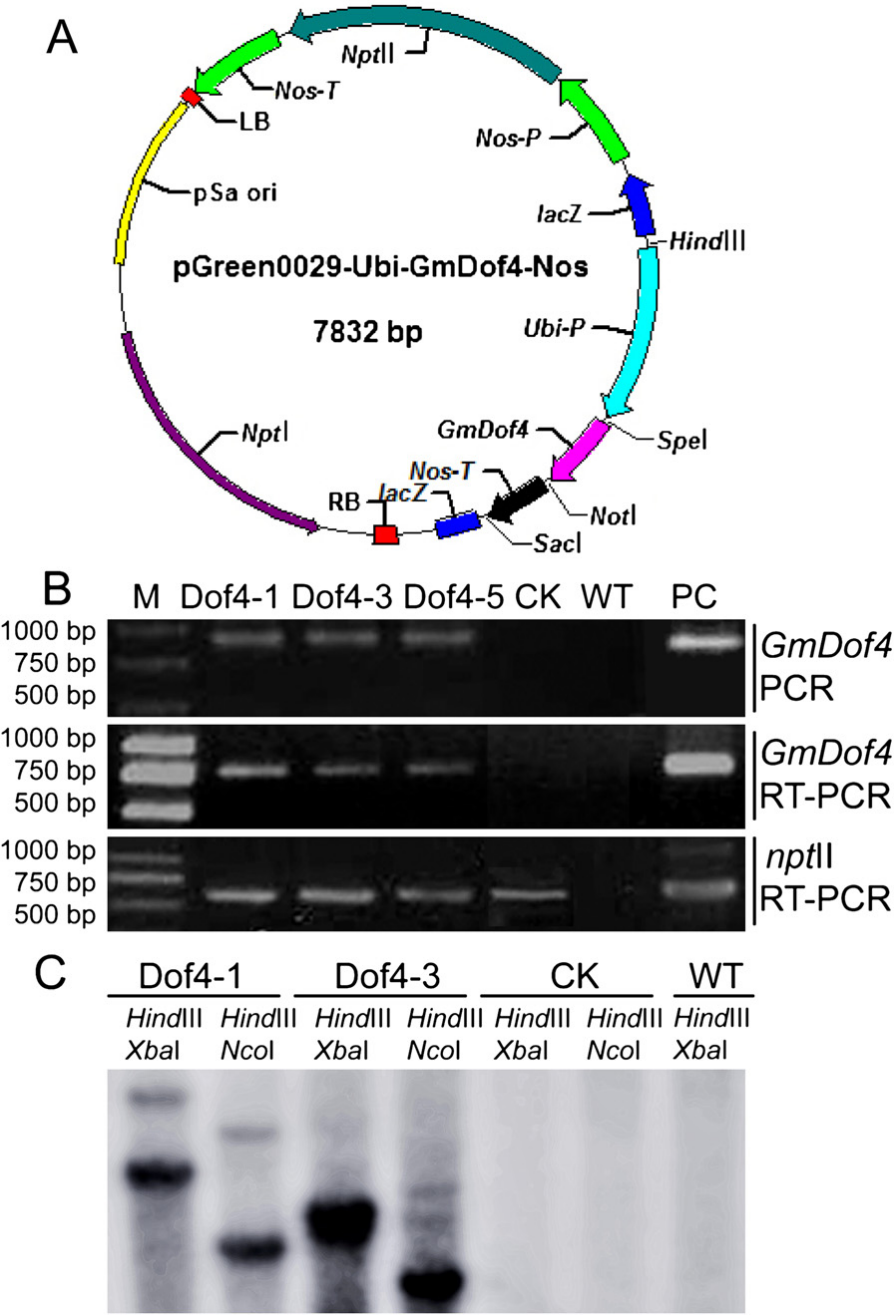
***GmDof4*** **transformation vector and detection of** ***GmDof4*** **and** ***npt*** **II in the transformants. (A)** A schematic map of the pGmDof4 plasmid. **(B)** PCR and RT-PCR detection of transgenic strains. Top panel: A 903 bp full length *GmDof4* cDNA sequence was amplified in transgenic strains by PCR; Middle panel: A 773 bp fragment of *GmDof4* cDNA was found in transgenic strains by RT-PCR; Bottom panel: A 623 bp fragment of *npt*II was found in transgenic strains by RT-PCR. **(C)** Southern blot detection of transgenic *GmDof4 Chlorella ellipsoidea*. The genomic DNA was digested with *Hind* III and *Xba* I or with *Hind* III and *Nco* I and then hybridized with a 545 bp fragment of the partial *GmDof4* gene, which was labelled with α-^32^P dCTP by Random Primer DNA Labeling Kit ver. 2.0 (Takara Biotechnology Co.,Ltd., Da Lian, China). Dof4-1, Dof4-3, and Dof4-5: different transgenic *GmDof4* strains. CK: pCK transgenic strains; PC: positive control; M: DNA molecular weight marker; WT: wild-type *Chlorella ellipsoidea*.

In this study, we transformed a plant expression vector, pGmDof4, into *C. ellipsoidea* by electroporation. pGmDof4 contained *GmDof4* under the control of the *Ubiquitin* promoter and the selection marker gene *npt*II (Figure 1A). Transformed cells were selected on Selenite Enrichment (SE) agar medium containing 30 mg/L G418. The selected clones were analyzed by PCR and RT-PCR (Figure 1B) and Southern blot (Figure 1C). The 903 bp *GmDof4*-specific band from pGmDof4 transgenic strains Dof4-1, Dof4-3, and Dof4-5 was amplified, but not those from control check (CK, the transgenic strain with the pCK that is identical to pGmDof4 but without the *GmDof4* expression cassette) and wild type (WT, non-transformed cells) (Figure 1B, top panel). Southern blot results indicated that *GmDof4* can be detected from the *GmDof4*-transformed cells with two bands in strain Dof4-1 and one thick band in strain Dof4-3 but no bands in the CK and WT (Figure 1C). This result suggested that *GmDof4* was inserted into the genome of *C. ellipsoidea* with two copies in Dof4-1 and at least one copy in Dof4-3, respectively. Furthermore, to verify the heterologous transcripts in *C. ellipsoidea*, the expression of *GmDof4* and *npt*II was detected by RT-PCR. In Figure 1B, the middle and bottom panels show that the *GmDof4* and *npt*II transcripts were both expressed in strains Dof4-1, Dof4-3, and Dof4-5, and only the *npt*II transcript was expressed in CK; neither was expressed in WT. Thus, we concluded that *Gmdof4* had been integrated into and is normally transcribed in the genome of the *C. ellipsoidea*.

### *GmDof4* expression in *C. ellipsoidea* does not affect the growth

**Figure 2:**
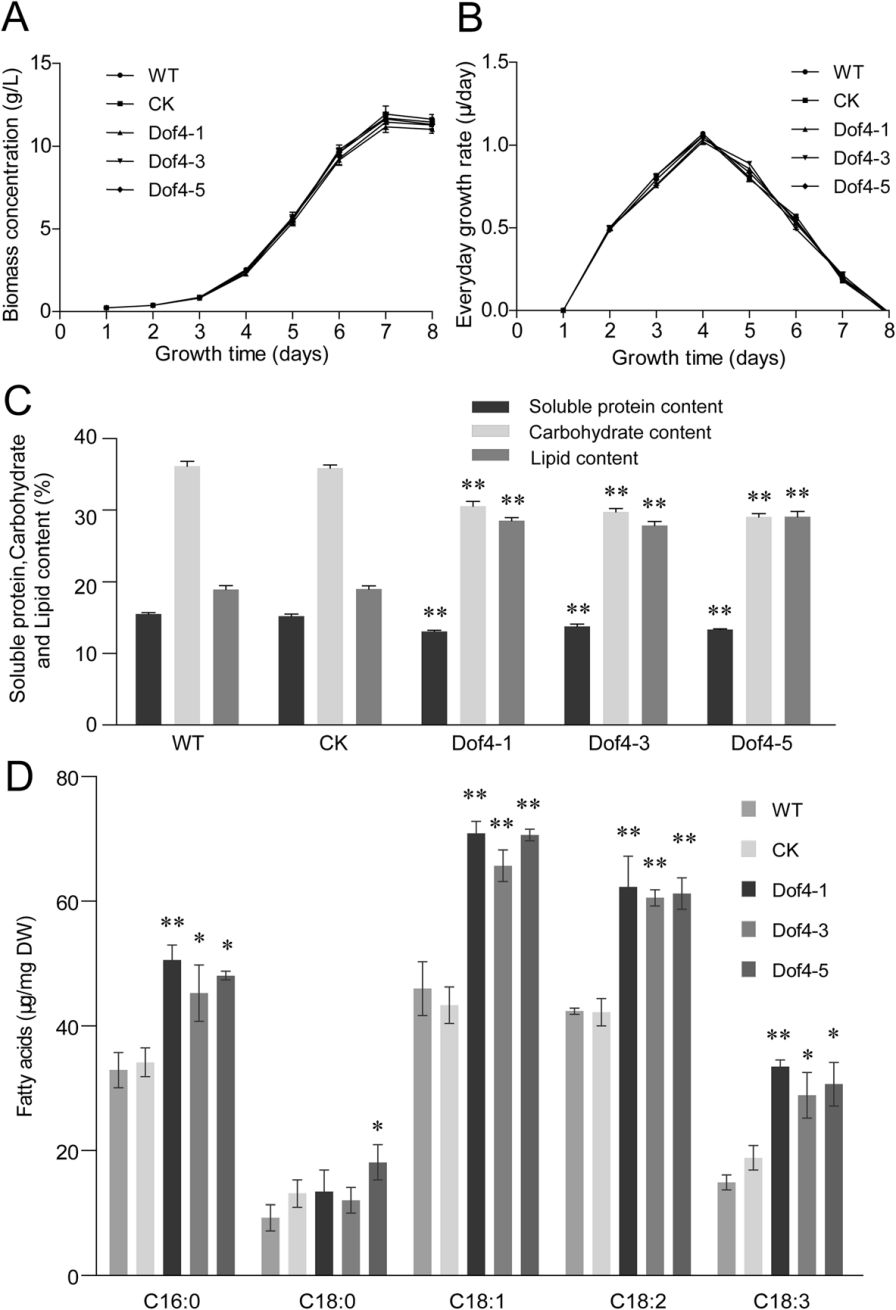
**Characterization of transgenic** ***C. ellipsoidea*** **expressing** ***GmDof4*** **under mixotrophic culture conditions. (A)** The growth curves of transgenic *GmDof4 Chlorella ellipsoidea* under mixotrophic culture conditions for eight days. **(B)** Growth rate of transgenic *GmDof4* strains compared with the control under mixotrophic culture conditions. **(C)** Total contents of the soluble protein, carbohydrate, and lipid of transgenic *GmDof4 Chlorella ellipsoidea* under mixotrophic culture conditions. The data represent the means ± SD of three replicate experiments and were analyzed by Student’s t-test (n = 3). Asterisks indicate a significant difference from pCK transgenic strains (**P <0.01). **(D)** The fatty acid content and composition in transgenic *GmDof4 Chlorella ellipsoidea*. The data represent the means ± SD of three replicate experiments and were analyzed by Student’s t-test (n = 3). *P <0.05; **P <0.01. CK, pCK transgenic strains; DW, dry weight; WT, wild-type *Chlorella ellipsoidea*.

The growth curves based on the biomass concentration and daily growth rate of the transgenic *C. ellipsoidea* cells under mixotrophic culture conditions (see Methods) were measured. Under this condition, the transgenic *GmDof4* strains, CK, and WT grew in the lag phase for the first two days, in the exponential phase from the third day to the seventh day, and in the stationary phase on the eighth day. The average biomass (dry weight) on the first day was 0.2319 to 0.2348 g/L, and the maximum biomass was 11.18 to 11.96 g/L on the seventh day (Figure 2A). During the exponential stage, the cell-doubling time was approximately 16 hours. The average daily growth rate of the different strains was approximately 0.55 μ/day (Figure 2B). The growth curve and the daily growth rate of the transgenic *GmDof4* strains, CK, and WT were similar, suggesting that the pGmDof4 transformation did not have deleterious effects on the growth of *C. ellipsoidea* cells.

### *GmDof4* expression in *C. ellipsoidea* increases the lipid content

The total soluble protein, carbohydrate, lipid content and composition, and content of fatty acids in the transgenic *Gmdof4* strains, CK, and WT were measured under mixotrophic culture conditions. The CK and WT strains had a similar total soluble protein content (average of 15.22% and 15.50%, respectively), carbohydrate content (average of 35.89% and 36.15%, respectively), and lipid content (average of 19.02% and 18.94%, respectively). Compared with CK, all three transgenic G*mDof4* strains showed the total soluble protein and carbohydrate content were significantly decreased, while the lipid content was significantly increased. The decrease in the total soluble protein in Dof4-1, Dof4-3, and Dof4-5 was 14.1%, 9.3%, and 12.4%, respectively; the decrease in the carbohydrate content was 14.9%, 17.1%, and 19.1%, respectively; and, the increase in the lipid content was 49.9%, 46.4%, and 52.9%, respectively (Figure 2C). The lipid productivity of the transgenic Dof4 strains was 0.45 to 0.47 g/L/d, significantly higher than the productivity of WT and CK (0.31 g/L/d; Table 1).

**Table 1:** **Lipid productivity of** ***Chlorella ellipsoidea*** **under mixotrophic culture conditions**

	**WT**	**CK**	**Dof4-1**	**Dof4-3**	**Dof4-5**
Lipid productivity (g/L/d)	0.31 ± 0.01	0.31 ± 0.01	0.45 ± 0.01*	0.45 ± 0.03*	0.47 ± 0.03*

The data represent the means ± SD of three replicate experiments and were analyzed by Student’s t-test (n = 3). *P <0.05. CK, pCK transgenic strains; WT, wild-type *Chlorella ellipsoidea*.

**Figure 3:**
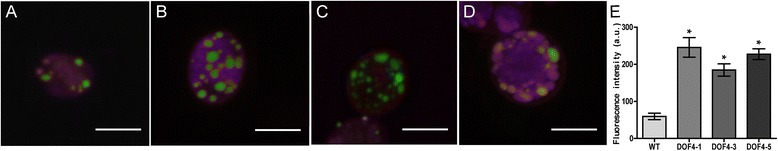
**Observation and determination of TAG droplets in** ***Chlorella ellipsoidea*** **under nutrient-limited conditions. (A)** WT strain; **(B)** Dof4-1; **(C)** Dof4-3; **(D)** Dof4-5; **(E)** Fluorescence intensity of *Chlorella ellipsoidea*. The data represent the means ± SD of three replicate experiments and were analyzed by Student’s t-test (n = 8). *P <0.05. TAG, triacylglycerol; WT, wild-type *Chlorella ellipsoidea*.

The lipid increase in the transgenic *GmDof4* strains could also be clearly observed by Nile red staining. More oil droplets accumulated in the transgenic *GmDof4* strains than in WT under nutrient-limited conditions (Figure 3A-D). Fluorescence was measured on a Varian 96-well plate spectrofluorometer, and the results showed that the transgenic *GmDof4* strains accumulate more TAG when compared to WT cells (Figure 3E).

Gas chromatograph-mass spectrometry (GC-MS) analysis indicated that the main fatty acid components of the transgenic *GmDof4* strains, CK, and WT were similar (Figure 2D); they consisted mainly of palmitic acid (C16:0), stearic acid (C18:0), oleic acid (C18:1), linoleic acid (C18:2), and alpha linolenic acid (C18:3). The abundance of the other fatty acids was too low to mention. With the exception of stearic acid (C18:0), the main fatty acid components of all three transgenic *GmDof4* strains showed significant increases. However, the relative compositions of total fatty acids were not significantly different from that in CK and WT (Table 2).

**Table 2:** **Lipid profile of** ***Chlorella ellipsoidea*** **under mixotrophic culture conditions**

**Fatty acids**	**Relative percentage**				
	WT	CK	Dof4-1	Dof4-3	Dof4-5
C16:0	22.64 ± 2.38	22.52 ± 2.63	21.91 ± 1.81	21.31 ± 2.68	21.01 ± 0.53
C18:0	6.37 ± 1.51	8.06 ± 1.76	5.83 ± 0.59	5.68 ± 1.65	7.92 ± 1.16
C18:1	31.61 ± 3.15	28.57 ± 3.32	30.74 ± 1.43	30.92 ± 2.07	30.88 ± 0.70
C18:2	29.14 ± 0.60	27.82 ± 2.54	27.01 ± 3.72	28.49 ± 1.08	26.77 ± 1.92
C18:3	10.23 ± 1.41	12.43 ± 1.27	14.52 ± 0.83	13.60 ± 1.01	13.42 ± 1.65

CK, pCK transgenic strains; WT, wild-type *Chlorella ellipsoidea*.

These results demonstrate that the expression of *GmDof4* enhances the lipid synthesis in *C. ellipsoidea* under mixotrophic culture conditions without any reduced biomass production.

### Altered gene expression in transgenic *GmDof4* cells under mixotrophic culture conditions

To understand the cellular mechanisms underlying the transformation of the pGmDof4 construct, transcriptome profiles for the transgenic *GmDof4* strain Dof4-1 and the CK samples were performed using the Illumina GAIIx platform. In total, 56,169 contigs with an average length of 441 bp were obtained. The size distribution for these contigs is shown in Additional file 1: Figure S1. The RNA-Seq data can be found in the Gene Expression Omnibus (GEO) library under the accession number [GSE:37473].

**Figure 4:**
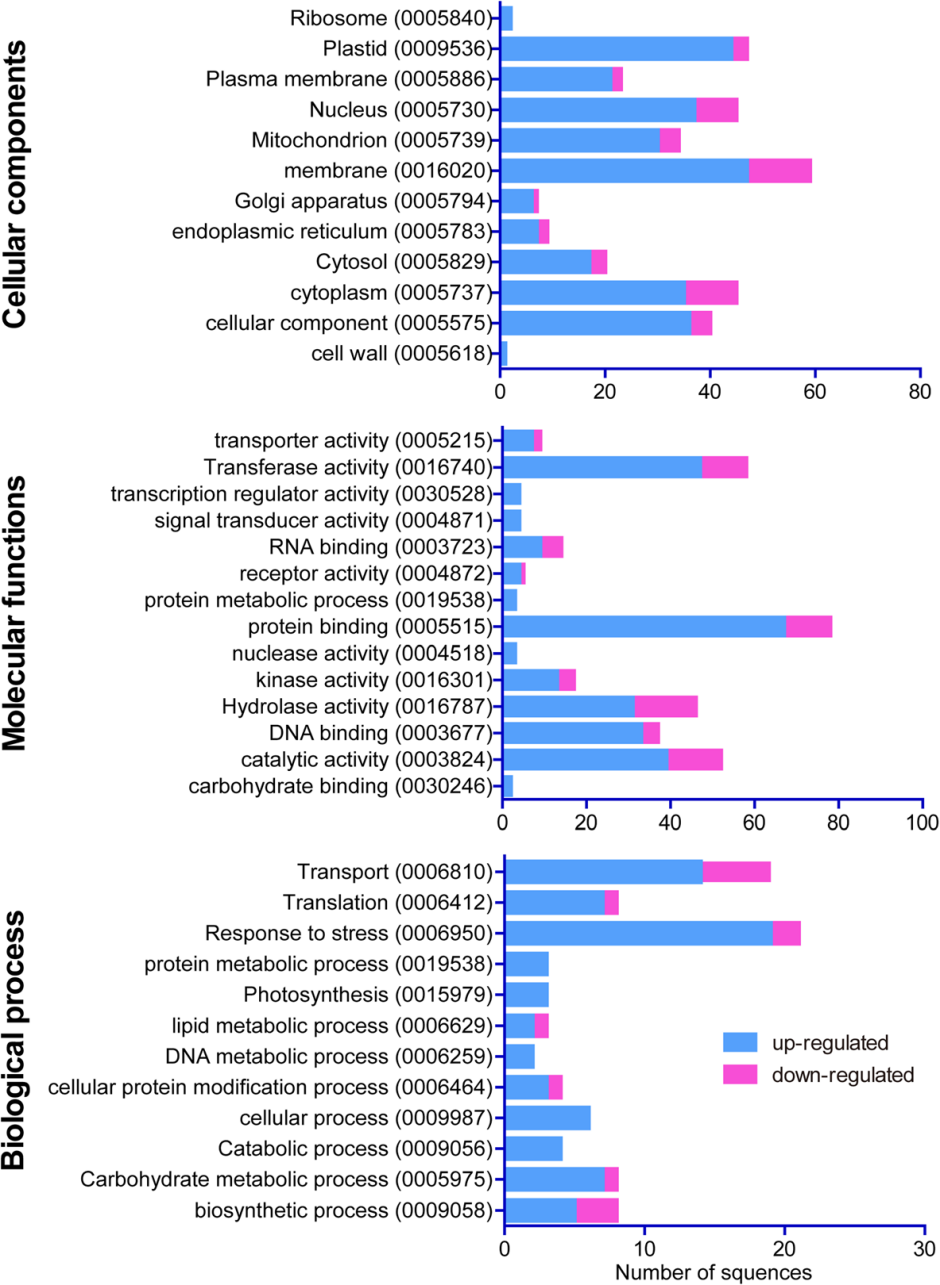
**The categories of** **GmDof4-regulated transcripts in transgenic** ***Chlorella ellipsoidea*** **.** The categories of the up- and downregulated transcripts were identified from RNA-seq analysis of the transgenic *GmDof4 Chlorella ellipsoidea* cells using gene ontology tools.

Our transcriptome analysis indicated that 1,076 contigs were differentially regulated (|log_2_ ratio| >1), of which 754 contigs, including 421 annotated and 333 unannotated contigs were significantly upregulated, and 322 contigs including 115 annotated and 207 unannotated contigs were significantly downregulated in the transgenic *GmDof4* samples. The regulated transcripts were annotated by gene ontology (GO) [24] and classified into hierarchic categories (Figure 4). The most abundant genes were found involved in the membrane and plastids in the cellular components category, protein binding and transferase activity in the molecular function category, and response to stress and transport in the biological process category. Then, we extracted those genes that were associated with fatty acid and lipid metabolism according to the GO annotation in an attempt to understand how *GmDof4* regulates the expression of those related genes.

The cumulative distribution of the log_2_ fold change (Additional file 2: Figure S2) of the transcripts that were associated with fatty acid and lipid metabolism showed a significant right shift, indicating that some transcripts were clearly upregulated. Table 3 lists the significantly regulated transcripts (|log_2_ ratio| >1) associated with lipid and fatty acid metabolism. Twenty transcripts were significantly upregulated, and two were downregulated significantly. These transcripts included 13 annotated transcripts involved in fatty acid and lipid biosynthesis: seven ACCases, three fatty acid synthases (FASs), one phosphatidylglycerol transferase, and two elongation of very long chain fatty acids proteins. There were three transcripts annotated as fatty acid and lipid transporter: one ATP-binding cassette (ABC) transporter and two Rft-1-domain-containing proteins. There were four transcripts involved in fatty acid and lipid catalysis: two phospholipases and two hydrolases. Interestingly, in all 20 upregulated transcripts, the top six (contig IDs: 56171, 80365, 101511, 71421, 67502, and 91597) transcripts and another transcript (contig ID: 59360) were all annotated as ACCases. These results indicated that most of the ACCase transcripts had been significantly upregulated by GmDof4 in *C. ellipsoidea*.

**Table 3:** **Transcripts associated with fatty acid and lipid metabolism that were regulated in transgenic** ***GmDof4 Chlorella ellipsoidea***

**Contig ID**	**Log**_**2**_ **fold change**	**Description**	**NCBI accession**	**Length**	**E value**
56171	8.41	acetyl-coenzyme a carboxylase	EIE18073.1	657	1.28E-74
80365	8.38	acetyl-coenzyme a carboxylase	EIE18073.1	600	3.76E-61
101511	8.14	acetyl-coenzyme a carboxylase	EIE18073.1	405	3.46E-38
71421	7.64	acetyl-coenzyme a carboxylase	EIE18073.1	764	4.48E-22
67502	7.19	acetyl-coenzyme a carboxylase	EIE18073.1	570	5.75E-74
91597	6.95	acetyl-coenzyme a carboxylase	EIE18073.1	1196	9.16E-41
83574	2.93	elongation of very long chain fatty acids protein 4	XP_002732296.1	1757	1.76E-36
69772	1.81	surface protein Sur1-like protein	EME32697.1	317	2E-25
85953	1.71	acetyl xylan esterase	EIE24862.1	785	5E-11
76019	1.58	phosphatidyl glycerophosphate synthase-like protein	XP_001699073.1	592	4E-15
91381	1.58	Rft-1-domain-containing protein	EIE21959.1	908	4E-47
85042	1.52	hypothetical protein COCSUDRAFT_48555	EIE20551.1	1569	2E-69
78075	1.44	Rft-1-domain-containing protein	EIE21959.1	930	9E-45
88610	1.28	Phospholipase/carboxylesterase	EIE25267.1	1196	2E-68
88508	1.21	fatty acid synthase	EIE23140.1	658	2E-83
86121	1.19	very-long-chain 3-ketoacyl-CoA synthase	EIE26326.1	1535	4E-129
59360	1.12	acetyl co-enzyme A carboxylase BC subunit	EIE26565.1	2230	0
83046	1.11	putative phosphatidylglycerol transferase	XP_002947306.1	789	2E-28
76421	1.07	fatty acid synthase	EIE23140.1	423	2E-83
82144	1.02	SGNH hydrolase	EIE19805.1	1721	1E-31
86271	−1	PREDICTED: fatty acid synthase isoform X5	XP_004861076.1	617	0.00003
59485	−1.7	phospholipase D alpha 2	NP_175666.1	3108	3E-134

TFs are able to recognize specific DNA sequences and establish protein-DNA and protein-protein interactions. They affect a large number of genes involved in multiple metabolic pathways, resulting in an integrated, simultaneously up- or downregulation of metabolites in these pathways. In Table 4, we listed 46 other significantly regulated transcripts (|log_2_ ratio| >2); they were divided according to GO categories into the metabolism (23), transport (6), binding (7), response to stress (4), and unclassified (6) categories. These results indicated that, in addition to the genes involved in lipid and fatty acid metabolism, many other genes with different functions were also strongly regulated by *GmDof4* in *C. ellipsoidea*.

**Table 4:** **Transcripts regulated in transgenic** ***GmDof4 Chlorella ellipsoidea***

**Function classification**	**Contig ID**	**Log**_**2**_ **fold change**	**Description**	**NCBI accession**	**Length**	**E value**
Metabolism	88349	5.10	flavodoxin	EIE22982.1	943	1.01E-46
	80694	3.09	flavin-containing monooxygenase	XP_001690742.1	318	1.43E-07
	81782	3.00	serine/threonine-protein kinase TOUSLED-like	XP_003545185.1	428	6.59E-13
	86913	2.93	tousled-like kinase 1	XP_002957155.1	656	1.09E-09
	83574	2.93	elongation of very long chain fatty acids protein 4	XP_002732296.1	1757	1.76E-36
	67904	2.92	sodium sulfate co-transporter	XP_001690490.1	1201	4.7E-101
	65347	2.84	sodium sulfate co-transporter	XP_001690491.1	1927	3.7E-136
	91555	2.76	serine threonine-protein kinase tousled-like 2	XP_002127920.1	360	3.24E-12
	81781	2.67	serine/threonine-protein kinase tousled-like	XP_003082583.1	848	1.1E-18
	82347	2.64	ATP-dependent RNA helicase	EIE18729.1	1571	1.27E-13
	85841	2.59	ferric-chelate reductase	EIE26078.1	1533	4E-50
	62217	2.47	hexokinase 2	EIE26809.1	3014	1.3E-80
	73871	2.37	cinnamyl alcohol dehydrogenase	EIE22193.1	1012	4.3E-54
	79933	2.36	cytosine-5 DNA methyltransferase	XP_003561341.1	340	8.97E-39
	81062	2.18	ubiquitin thioesterase	EIE18214.1	1460	6.56E-79
	89786	2.05	transcription initiation factor TFIID subunit 6-like	XP_003577929.1	345	6.85E-26
	93291	−5.51	cytochrome P450	EIE19060.1	527	0.000213
	83571	−2.59	alpha/beta-hydrolase	EJD50872.1	940	1.91E-29
	72299	−2.53	endoribonuclease l-psp	YP_002251050.1	855	1.99E-11
	68852	−2.43	fad linked oxidase domain protein	YP_001699676.1	1274	1.11E-15
	68856	−2.26	FAD-dependent oxidoreductase	YP_001252809.1	776	6.45E-32
	76145	−2.23	serine threonine protein kinase 9	EIE18318.1	893	1.68E-32
	59899	−2.12	hydroxydechloroatrazine ethylaminohydrolase	EIE19019.1	1628	1.6E-124
Transport	58206	3.41	Snf7-domain-containing protein	EIE26516.1	765	7.21E-20
	56450	2.54	sugar transport protein	EIE22371.1	2478	3.4E-116
	62118	2.19	sugar transport protein	EIE26164.1	2507	1.23E-97
	70774	−4.03	hypothetical protein CHLNCDRAFT_145405	EFN56009.1	1016	0.000108
	62487	−3.06	uric acid-xanthine permease	XP_001690343.1	959	4.3E-69
	80480	−2.1	purine permease	XP_002152129.1	542	2.09E-24
Binding	76355	3.59	zinc finger protein zf1	XP_003081226.1	1037	3.55E-11
	59250	3.58	f-box protein	YP_007198.1	975	1.96E-16
	79167	3.09	p115-like protein	NP_566820.1	1230	1.48E-49
	92615	3.08	argonaute protein group	EIW84641.1	417	2.61E-25
	77846	2.72	f-box protein/LRR-repeat protein	EIE24729.1	1000	2.73E-11
	84262	2.62	hsp100 family	EIE26713.1	2508	0
	99456	2.08	Zinc-finger protein	XP_002007677.1	327	0.000952
Response to stress	86306	3.58	heat shock protein hsp20	EFN56197.1	939	3.27E-13
	86715	3.03	kda class i heat shock protein 1	EFN56197.1	1089	9.83E-15
	72405	2.36	heat shock transcription factor 1	XP_001694420.1	1239	4.37E-25
	86717	2.28	heat shock protein 17	EFN56197.1	746	9.6E-22
Unclassified	58160	2.79	vesicle docking protein	XP_003079367.1	1828	2.61E-14
	87605	2.25	hypothetical protein CHLNCDRAFT_32684	EFN52166.1	506	3.29E-20
	89602	2.06	predicted protein	XP_001689881.1	312	6.48E-05
	81453	−2.5	FkbM family methyltransferase	ZP_08847471.1	1353	4.94E-25
	73041	−2.23	glutathione S-transferase related protein	ABL97197.1	939	0.000213
	56771	−2.05	periplasmic l-amino acid catalytic subunit	AAB97101.1	2207	4.8E-66

The transcripts associated with fatty acid and lipid metabolism were excluded.

### Validation of RNA-seq analysis

**Figure 5:**
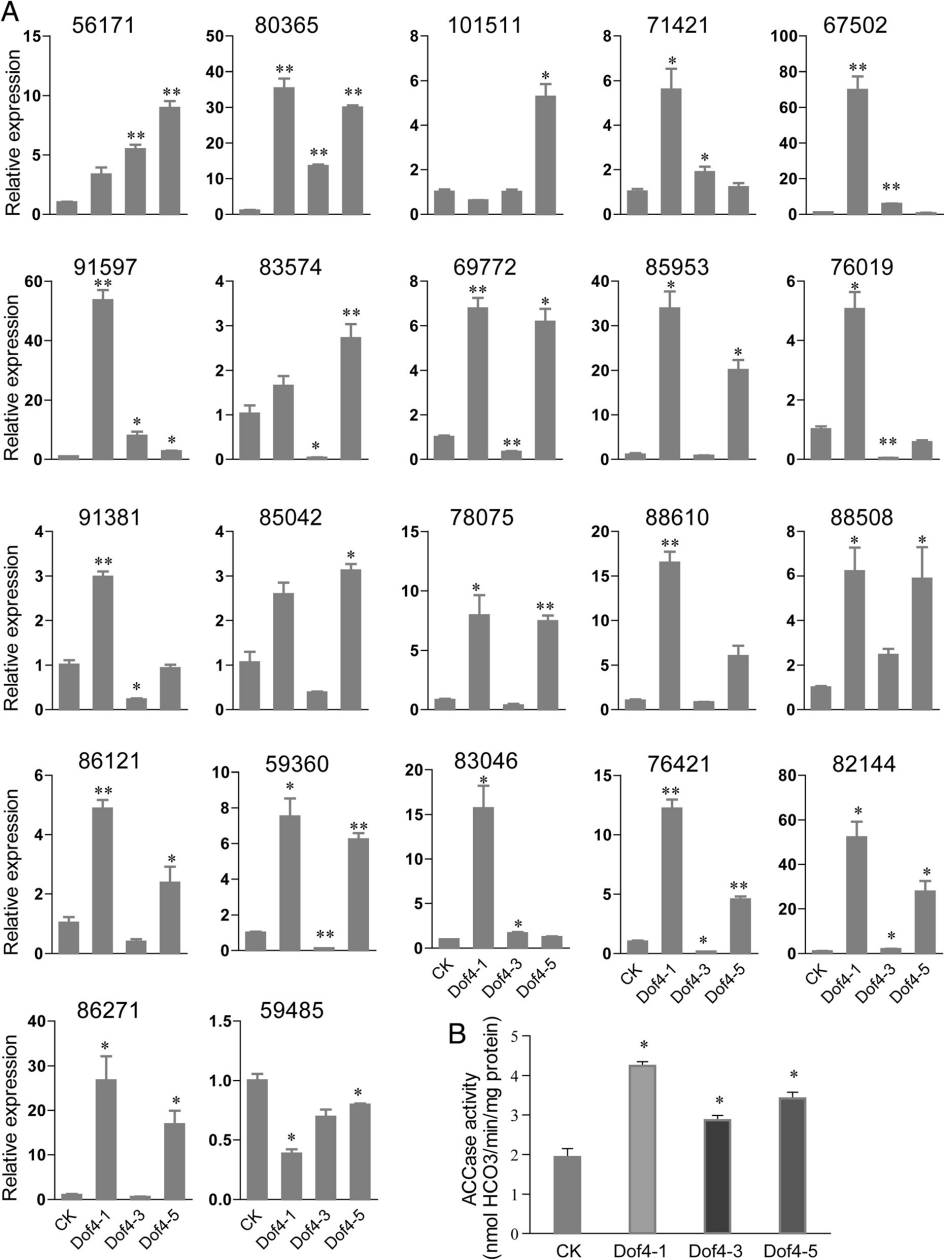
**Validation of RNA-seq by qRT-PCR and an enzyme activity assay. (A)** Gene expression detected by qRT-PCR in *Chlorella ellipsoidea*. The relative expression of 20 upregulated and two downregulated genes related to lipid and fatty acid metabolism was determined by qRT-PCR. The data represent the means ± SD of three replicate experiments and were analyzed by Student’s t-test (n = 3). *P <0.05; **P <0.01. **(B)** ACCase activity in crude cell extracts of CK and transgenic *GmDof4* strains. The data represent the means ± SD of three replicate experiments and were analyzed by Student’s t-test (n = 3). *P <0.05. ACCase, acetyl-coenzyme A carboxylase; CK, pCK transgenic strains.

The relative expression levels of 22 significantly regulated transcripts that are associated with lipid and fatty acid metabolism were analyzed by qRT-PCR analysis (Figure 5A). The *Chlorella* 18S rRNA gene was used as an internal control. Three independent transgenic strains were analyzed. The regulation patterns of 20, 10, and 19 transcripts were confirmed by qRT-PCR in Dof4-1, Dof4-3, and Dof4-5, respectively. The results indicated that the expression levels of most of the transcripts detected by qRT-PCR were consistent with those detected by Solexa RNA-seq analysis. However, there were more variations among the different transgenic strains. We also tested the expression of the GmDof4 transcript; there was no expression in CK, and the GmDof4 transcript level in Dof4-1 was higher than that in Dof4-3 and Dof4-5, but similar to that in Dof4-3 and Dof4-5 (Additional file 3: Figure S3). The variation of the expression levels of the transcripts in different strains may be due to the difference in the integration sites, copy numbers, or other reason caused by nuclear transformation.

### ACCase activity is enhanced in transgenic *C. ellipsoidea* cells

The ACCase activity was assayed in the transgenic *GmDof4* strains and CK (Figure 5B). The ACCase activity of Dof4-1, Dof4-3, and Dof4-5, with average values of 4.25, 2.87, and 3.42 nmol HCO_3_/min/mg protein, respectively, were significantly higher than that of CK (average value of 1.93 nmol HCO_3_/min/mg protein). The fact that the ACCase activity was increased was consistent with the additional transcript numbers indicated by the RNA level in transgenic *GmDof4* strains.

## Discussion

### *GmDof4* expression in *C. ellipsoidea* increases the lipid content but does not affect the growth

Microalgae are an attractive renewable biodiesel feedstock because their productivity is 20 to 40 times higher than that of oil crops [25]. Rapidly growing algal cells, which are suited for large-scale production in highly variable outdoor conditions, contain lower amounts of lipid (≤20% of dry weight), whereas algal cells with high lipid contents (40 to 50% of dry weight) grow very slowly [26]. Increasing the lipid content of rapidly growing microalgae is a desirable approach to producing biodiesel by cell culture. To date, significant advances in microalgal genomics have been achieved [6,27], and two reports of enhanced lipid content of diatoms due to genetic engineering have been published [16,17].

In this research, we transferred *GmDof4* into the unicellular eukaryotic organism *C. ellipsoidea,* and we found that the total lipid content of *GmDof4* transgenic *C. ellipsoidea* was increased by 46.4 to 52.9% relative to that of CK under mixotrophic culture conditions. In contrast, the overexpression of *GmDof4* in *Arabidopsis* resulted in an increase in the seed lipid content by 22% over that of wild-type plants [23]. To our knowledge, this is the first report of significantly increasing the lipid content of *Chlorella* by genetic engineering. Along with the clearly increased total lipid content, the total soluble protein and carbohydrate content were significantly decreased in *GmDof4* transgenic *C. ellipsoidea* expressing GmDof4. These results indicated that there is a balancing system that regulates metabolism and energy exchange in transgenic *C. ellipsoidea* cells.

However, we found that the growth curve and the growth rate of transgenic *GmDof4 C. ellipsoidea* were not different from those of CK and WT under mixotrophic culture conditions in the Endo medium (Figure 2A,B). This result suggested that the *GmDof4* expression in *C. ellipsoidea* did not have a deleterious effect on the growth of host cells although the *GmDof4* originated from a higher plant. Using TFs to engineer microalgae for increased the lipid content without decreasing the growth rate of host cells is a significant advance. Certainly, scaled-up culture must still to be tested for biodiesel production.

To determine whether more carbon was entering the cell via mixotrophic growth, we measured the growth rate and lipid content of the transgenic *C. ellipsoidea* expressing *GmDof4* under an autotrophic culture condition. This condition did not significantly change the growth, but the lipid productivity was still increased in transgenic *GmDof4* strains (Additional file 4: Figure S4; Additional file 5: Table S1). These results indicated that the increased lipid content might result from the conversion of internal source of carbon rather than an exogenous carbon input.

We also found that all three transgenic *GmDof4* strains showed significant increases in the absolute content of C18:1, C18:2, C18:3, and C16:0 fatty acids compared with those in CK, but there was no significant difference in the relative content of these main fatty acids under mixotrophic culture conditions (Table 2). In contrast, the relative level of C18:2 was significantly decreased in *GmDof4* transgenic *Arabidopsis* plants [23]. This result suggested that the expression of *GmDof4* in different organisms may result in a shift of certain fatty acid component.

### GmDof4 significantly regulated the gene expression of *C. ellipsoidea*

*GmDof4* is a flower/pod-specific gene and can increase the lipid content and weight of *Arabidopsis* seeds by regulating the gene expression network involved in lipid biosynthesis. In *Arabidopsis*, the expression of 104 genes was upregulated and that of 64 genes was downregulated by GmDof4 [23]. Our results indicated that the expression of 754 genes was significantly upregulated and that of 322 genes was significantly downregulated in transgenic *GmDof4 C. ellipsoidea*. In this regulatory network, the transcripts of ACCase became the center of attention.

ACCase catalyzes the first key step of fatty acid biosynthesis in a two-step reaction that results in the conversion of acetyl-CoA to malonyl-CoA. Most plants have two forms of ACCase: a homomeric form in the cytosol that is composed of a single large polypeptide catalyzing the individual carboxylation reactions, and a heteromeric form in plastids that is composed of four subunits [biotin carboxylase (BC), biotin carboxyl carrier protein (BCCP), α-carboxyl transferase (α-CT), and β-carboxyl transferase (β-CT)] [28,29]. Guarnieri *et al.* [30] reported that the ACCase abundance was upregulated approximately two-fold in *Chlorella vulgaris* strain UTEX 395 under nitrogen-depleted conditions. Using microarray and DNA-binding analysis, the GmDof4 protein was previously shown to activate *accD* expression by directly binding the promoter of *accD* at position −287 to −274 in transgenic *Arabidopsis* [23]. Using RNA-seq and qRT-PCR, we found that seven transcripts encoding ACCase were significantly upregulated. In particular, the top six up-regulated transcripts (contig IDs: 56171, 80365, 101511, 71421, 67502, and 91597) were all annotated as ACCase. Transcriptome analysis and conservative binding region comparisons [31] revealed that the genes that encode both forms of ACCase in transgenic *GmDof4 C. ellipsoidea*. Contig ID 59360 is a part of heteromeric form of ACCase biotin carboxylase (BC) subunit gene that was significantly upregulated in transgenic *GmDof4 C. ellipsoidea* (Table 3), whereas contig ID 56174 is a part of a homomeric form of the ACCase BC subunit gene that was not significantly regulated in transgenic *GmDof4 C. ellipsoidea*. However, we could not identify the form of the other six upregulated ACCase genes because the sequence information was incomplete. Nevertheless, it is certain that GmDof4 protein strongly upregulates ACCase in transgenic *C. ellipsoidea* cells. Using an enzyme activity assay analysis, we confirmed that the ACCase activity was significantly increased in transgenic *C. ellipsoidea* cells in the exponential phase. However, the detailed mechanism of the regulation of ACCase enzyme activity by the GmDof4 protein in *C. ellipsoidea* must still be investigated. Future studies will aim at obtaining the GmDof4-binding regions of ACCase genes, which are usually the promoter regions of these genes.

In addition to ACCase genes, FAS gene, and phospholipase D gene were significantly regulated in transgenic *GmDof4 C. ellipsoidea* in the exponential phase. FAS is a multi-enzyme system that catalyzes fatty acid synthesis. Its main function is to catalyze the synthesis of palmitate from acetyl-CoA and malonyl-CoA, in the presence of reduced nicotinamide adenine dinucleotide phosphate (NADPH), into long chain saturated fatty acids. Phospholipase D is an enzyme that catalyzes the hydrolysis of phosphatidylcholine to form the phosphatidic acid (PA) that is involved in lipid degradation [32]. These results demonstrated the pivotal role of the GmDof4 protein in lipid and fatty acid metabolism.

The Dof proteins are thought to regulate the expression of particular genes via binding to the promoter or via specific protein-protein interactions. In our study, we classified the significantly regulated genes from transcriptome analysis into binding (seven upregulated genes), metabolism (22 upregulated genes, and seven downregulated genes), response to stress (four upregulated genes), and transport (three upregulated genes and three downregulated genes) categories using GO tools. Some of the genes regulated by the GmDof4 protein in transgenic *Arabidopsis* and *C. ellipsoidea* are the same, such as ACCase beta subunit, glutathione S-transferase, and cytochrome P450, but there are still many different genes that are regulated in different species, such as 12S seed storage protein (CRA1) and male sterility MS5 family protein. These differences could be caused by the great genomic diversity between higher plant and unicellular green alga. In short, GmDof4 may play a comprehensive role in the increase total lipid content and may regulate genes related to lipid, fatty acid, protein, and carbohydrate metabolism in transgenic *C. ellipsoidea* cells. Certainly, the network of target genes regulated by GmDof4 in *C. ellipsoidea* needs to be characterized further in detail.

Although complete genome sequences from the unicellular green algae *Chlorella variabilis* NC64A have been obtained [33], little is known about the transcription factors involved in lipid metabolism. Recent advances in systems biology analyses of unsequenced microalgae could provide new tools to accelerate the production of next-generation biodiesel [34]. Moreover, using transcriptomics and proteomics to examine the triacylglycerol biosynthetic pathway in *C. vulgaris* will greatly accelerate the commercialization of microalgae-derived biodiesel [30,35]. These efforts establish a foundation for elucidating the key components of microalgal lipid productivity enhancement. Our results suggested that a TF from higher plants could be used to improve the lipid content of *C. ellipsoidea* and, most likely, other species*.*

## Conclusions

The hetero-expression of a gene for a transcription factor, *GmDof4*, from soybean (*G. max*) can significantly increase the lipid content while not affecting the growth rate of *C. ellipsoidea* under mixotrophic culture conditions. The increase of lipid content could be attributed by *GmDof4* gene regulatory network that enhances ACCase gene expression and enzyme activity in the transgenic cells. Our research provides a new way to increase the lipid content of microalgae by introducing a specific transcription factor to microalgae strains that can be used by the biofuel industry.

## Methods

### Strains and culture conditions

The *C. ellipsoidea* cells used in this study were grown in Endo medium [36] for the mixotrophic culture and in KNOP medium [37] for the autotrophic culture in a rotary shaker (DZ-900, Zhongkepusen Co., Ltd., Beijing, China), 200 rpm at 25°C under illumination (100 μmol photons/m^2^/s).

### Cloning of *GmDof4* cDNA

The cDNA of *GmDof4* was amplified from leaves of soybean cultivar Kefeng 1 using primers P1 and P2 (Additional file 6: Table S2), which were designed according to the published *GmDof4* cDNA sequence (accession number: [GenBank: DQ857254.1]). The *GmDof4* cDNA was cloned into a T-vector (pEASY-Blunt Cloning Vector, TransGen Biotech. Ltd., Beijing, China) resulting in pEB-GmDof4.

### Construction of the *Gmdof4* plant expression vector

The Nopaline synthase (nos) terminator was amplified by PCR with primers P3 and P4 (Additional file 6: Table S2) from plasmid vector pGreen0029 (Biotechnology and Biological Sciences Research Council, BBSRC, Wiltshire, United Kingdom) and was cloned into the site between *Not* I and *Sac* I in pGreen0029, resulting in an intermediate vector pGreen0029-Tnos. The ubi promoter region from maize [38] was amplified by PCR from plasmid vector pBI221 (Clontech Laboratories Inc., Mountain View, United States) with primers P5 and P6 (Additional file 6: Table S2) and cloned into the site between *Hind* III and *BamH* I in pGreen0029-Tnos, resulting in a vector pGreen0029-Pubi-Tnos (pCK) that confers resistance to the aminoglycoside antibiotics, such as G418. The DNA fragments encoding GmDof4 were obtained by digesting the pEB-GmDof4 with *Spe* I and *Not* I, and the fragments were then inserted into the site between *Spe* I and *Not* I in the plastid pCK at the downstream of the ubi promoter, resulting in vector pGreen0029-Ubi-GmDof4-Nos (pGmDof4).

### Transformation of *C. ellipsoidea*

*C. ellipsoidea* was transformed according to a previously published method [39]. Briefly, cells were cultured to the logarithmic phase in Endo medium (10 mL, containing approximately 10^7^ cells/mL), collected by centrifugation and resuspended in 10 mL of a solution of 0.2 M mannitol and 0.2 M sorbitol (Sigma Aldrich, St. Louis, United States). The resuspended cells were kept on ice for 1 hour, centrifuged and resuspended in electroporation buffer (0.08 M KCl, 0.005 M CaCl2, 0.01 M HEPES, 0.2 M mannitol, and 0.2 M sorbitol) at a concentration of approximately 10^8^ cells/mL and then immediately mixed with a final concentration of 20 μg/mL pGmDof4 plasmid, a final concentration of 10 μg/mL plasmid pSoup, and 25 μg/mL salmon sperm DNA (Invitrogen, Carlsbad, CA, United States). In total, 0.4 mL of the cell suspension was removed, kept on ice for 5 to 10 minutes, and subsequently used for transformation. The cells were transformed with a Baekon 2000 (Baekon Co., California, United States) electroporation device using 6 kV of between 0.001 and 0.02 second pulse duration, 2^10^ pulse frequency and 2 mm pulse distance for 100 cycles. After electroporation, the cells were screened using SE agar [40] selection medium containing 30 mg/L G418. The individual clones on the selection medium could be obtained after 25 to 30 days and they were continuously selected once per month for at least eight times. The selected individual strains were subcultured in SE liquid medium containing 15 mg/L G418.

### Transgenic cells identification by PCR and RT-PCR

The individual clones grown on the selection medium were subcultured in SE liquid medium with 15 mg/L G418. The cells at a cell density of approximately 1× 10^8^ cells/mL were collected by centrifugation at 12,000 g for 10 minutes. The DNA was extracted using the hexadecyl trimethyl ammonium bromide (CTAB) method. *GmDof4* was detected by PCR using primers 1 and 2 (Additional file 6: Table S2). The PCR products were analyzed by electrophoresis on a 1% agarose gel (Gene Company Ltd., Hong Kong, China) and sequenced by SinoGenoMax Co., Ltd., (Beijing, China). For RT-PCR detection, the RNA was isolated from the cells using the guanidinium thiocyanate-phenol-chloroform extraction procedure [41]. The expression of *GmDof4* and *npt* II was detected by RT-PCR using primers 9 and 10 and primers 11 and 12 (Additional file 6: Table S2), respectively, which were designed on the basis of coding region of the *GmDof4* and *npt* II genes. RT-PCR products were analyzed by electrophoresis on a 1% agarose gel and by sequencing.

### Southern blot analysis

Approximately 20 μg of genomic DNA of PCR identified transgenic clones was separately digested with different restriction endonucleases, separated on a 0.8% agarose gel, blotted onto a nitrocellulose membrane (Amersham Bioscience, Little Chalfont, United Kingdom) and hybridized with the probe that was amplified from the vector pGmDof4 by the primers P7 and P8 (Additional file 6: Table S2), which were designed on the basis of part of the coding region of the *GmDof4* gene. The probe was labeled with dCTP α-^32^P using a random primer labeling kit (Takara Biotechnology Co., Ltd., Dalian, China). The hybridization was performed according to the description by Sambrook and Russell [42].

### Biomass analyses

All the biomass analyses were performed using transgenic and wild-type *C. ellipsoidea* strains grown in 100 mL of Endo medium and grown in KNOP medium for autotrophic culture in a rotary shaker at 25°C under illumination (100 μmol photons/m^2^/s).

The *C. ellipsoidea* biomass concentration (w/v) is equivalent to a specific value of the cell dry weight (DW) that was determined by OD_540_ according to the following empirical formula:

1 $$ DW\ \left(g/L\right) = \left(O{D}_{540} + 0.0097\right)/0.4165 $$

The specific growth rate of *C. ellipsoidea* was calculated according to the equation [43]:

2 $$ SGR\ \left(\mu / day\right) = ln\left({X}_2\hbox{--} {X}_1\right)/\left({t}_2\hbox{--} {t}_1\right) $$

where X_1_ is initial biomass concentration, X_2_ is ending biomass concentration, and (t_2_–t_1_) is elapsed time.

### Measurement of the soluble proteins, carbohydrate, lipid content, and the fatty acid composition

Transgenic and wild-type *C. ellipsoidea* strains were cultured in Endo medium in a rotary shaker at 25°C under illumination (100 μmol photons/m^2^/s) for seven days. The biomass was collected to measure the soluble proteins, carbohydrate, lipid content, and the fatty acid composition.

### Detection of proteins and carbohydrate

Calibration curves were generated for each of the cellular constituents using D-glucose for carbohydrate (Beijing Chemical Works, Beijing, China). The carbohydrate content was analyzed based on the procedure published by Miao and Wu [44]. In short, 0.1 g of dried algal pellet was acidified by adding 20 mL of 2.5 M HCl (Beijing Chemical Works, Beijing, China). The acidified solution was then hydrolyzed at 100°C for 30 minutes and neutralized to pH 7. The volume was adjusted to 100 mL. The filtered sample was subjected to a 3,5-dinitrosalicylic acid (DNS) assay. Proteins were extracted following the procedure of Rausch [45] and were quantified using the Bradford method [46].

### Lipid content measurement

Lipid extraction was performed by the Soxhlet method that was similar to the procedures reported by Folch *et al*. [47]. Briefly, the total biomass was harvested by low-temperature centrifugation, washed with distilled water, frozen at −20°C, freeze-dried for 24 hours, and then gravimetrically determined. The cell paste was dried at 40°C for 12 hours and then the dry biomass was ground into powder. Accurately weighed 0.5 g of the powder was mixed with 125 mL trichloromethane:methanol (2:1, v/v) solvent (Beijing Chemical Works, Beijing, China). The extraction was performed at 100°C for 4 hours to ensure maximum recovery. The lipid was recovered using a rotating vacuum evaporator.

### Lipid productivity calculation

Daily lipid productivity was calculated using the equation:

3 $$ Daily\  lipid\  production\ \left(g\  lipid/\ L/\  day\right) = DW \times lipid\  content/\  day $$

where DW is algal dry weight (g /L), lipid content is %DW, and day is growth period.

### Fatty acid composition detection

The fatty acid composition was qualitatively and quantitatively determined using a TurboMass gas chromatography mass spectrometer (PerkinElmer, Massachusetts, United States) with a capillary column (BPX-70, 30 m × 0.25 mm × 0.25 μm) using the method described by Kattner and Fricke [48] and Song *et al*. [49]. Briefly, cellular fatty acid was extracted from 50 mg of *C. ellipsoidea* powder in 3 mL of 7.5% (w/v) potassium hydroxide in methanol for saponification at 70°C for 3 hours. After the pH was adjusted to 2.0 with hydrochloric acid, the fatty acids were subjected to methyl esterification with 2 mL of 14% (w/v) boron trifluoride in methanol (Beijing Chemical Works, Beijing, China) at 70°C for 1.5 hours. Then 1 mL of 0.9% (w/v) sodium chloride was added and was mixed well. Subsequently, fatty acid methyl esters (FAMEs) were extracted with 4 mL of hexane (Beijing Chemical Works, Beijing, China). The upper phase was removed to a second tube, dried under N_2_ and dissolved in acetic ether. FAMEs were analyzed and identified by the comparison of their peaks with a known internal standard 17:0 FAME (Sigma Aldrich, St. Louis, MO, United States).

### Illumina-based RNA-seq analysis

#### Library construction and sequencing

The transgenic *GmDof4* strain Dof4-1 and the CK (pCK transgenic strain) were cultured in liquid antibiotic-free Endo medium at 25°C under illumination for 120 hours. Three physical duplicates were cultured simultaneously. Cells at a concentration of approximately 1 × 10^7^ cells/mL were collected for library construction and sequencing. In this step we mixed the three physical duplicates as a hybrid sample for RNA isolation. Poly (A) RNA was isolated from 10 μg of total RNA and the purified mRNA was first fragmented into small pieces (100–800 bp, main band 300–600 bp) at 94°C for exactly 1.5 minutes. Then, double-stranded cDNA was synthesized, and the synthesized cDNA was subjected to end-repair and phosphorylation. Illumina adapters were ligated to the ends of these 3′-adenylated cDNA fragments. To select a size range of templates for downstream enrichment, the products of ligation reaction were purified on a 2% TAE-agarose gel. A range of cDNA fragments (350–450 bp) was excised from the gel. Fifteen rounds of PCR amplification were performed to enrich the purified cDNA template. The cDNA library was sequenced on a SE flow cell using Illumina Genome Analyzer IIx (Illumina, San Diego, California, United States). Finally, a total of 70,763,828 raw reads with a length of 80 bp were generated from two GA IIx single-end lanes.

### Assembly

We merged all the sequences from three libraries (another transgenic library was added for assembly) to use as the input data for assembly to obtain a better assembly result. Using SOAPdenovo [50] with the parameters “*-K31–d3–R*”, 56,169 contigs with an N50 contig size of 1,029 bp were obtained.

### Detection of differentially expressed gene

Contigs with a length of at least 300 bp were used as reference sequences. To detect the differentially expressed genes, we first mapped the short reads to the reference genes using the Burrows-Wheeler alignment tool (BWA) program [51] with default parameters. The number of reads mapped to each reference gene was counted as the expression level and the fold-change was calculated after normalization of the total reads for the two libraries. DEGseq detected 1,076 differentially expressed genes with a fold-change higher than two and a false discovery rate less than 0.001.

### Annotation

For validation and annotation of the assembled contigs, a sequence similarity search was conducted against a non-redundant protein database using the BLASTx algorithm with an E value threshold of 10^−3^. The results revealed that out of 13,566 contigs, 7,559 (55.72%) showed significant similarity to known proteins in the non-redundant (Nr) database. Contigs with a similarity greater than the threshold were annotated using GO according to the molecular function, biological process, and cellular component ontologies (http://www.geneontology.org) by the Blast2GO program [52].

### Quantitative real-time PCR (qRT-PCR) assay

The transgenic *GmDof4* strains and the CK were cultured in liquid antibiotic-free Endo medium at 25°C under illumination for 120 hours. Then, the total RNA was isolated using EASYspin plant RNA isolation kits (Aidlab Biotechnologies Co., Ltd, Beijing, China). qRT-PCR was performed on a LightCycler® 480 Real-Time PCR System (Roche Applied Science, Mannheim, Germany) using LightCycler® 480 SYBR Green I Master (Roche Applied Science, Mannheim, Germany) according to the manufacturer’s instructions: 1 cycle of 95°C for 30 seconds and then 40 cycles of 95°C for 10 seconds, followed by 55°C for 10 seconds, and 72°C for 20 seconds. The primers used for qRT-PCR are shown in Additional file 6: Table S3. Furthermore, the 20 μL reaction solution for real-time PCR was composed of 5 μL of cDNA, a 0.5 μM final concentration of each primer, and 10 μL of 2× real-time PCR Master Mix (Roche Applied Science, Mannheim, Germany). To normalize the amount of transcripts in each sample, the relative abundance of 18S rRNA was also determined and was used as the internal standard (forward primer: 5′-CTTGTAAACCGCGTCGTGATG-3′, reverse primer: 5′-GACGTAATCAACGCGAGCTGAT-3′). The gene expression data was analyzed using the 2^-ΔΔCt^ method [53].

### ACCase activity assay

The ACCase assay of transgenic *GmDof4* cells and CK was determined according to a previously published method [54]. The transgenic *GmDof4* strains and CK were cultured in liquid antibiotic-free Endo medium at 25°C under illumination for 120 hours. Then, crude cell extracts were prepared by grinding fresh cells on ice in 2 volumes (w/v) of 50 mM Tris-Cl, pH 7.5, 100 mM potassium chloride, 5 mM magnesium chloride, 1 mM dithiothreitol, 0.1% TritonX-100, 10% (v/v) glycerol, and 0.2 mM Phenylmethanesulfonyl fluoride (Sigma Aldrich, St. Louis, MO, United States). The homogenates were centrifuged for 5 minutes at 3,000 × g and were desalted using PD-10 columns. The protein content was determined by the Bradford assay using bovine serum albumin (Sigma Aldrich, St. Louis, MO, United States) as a standard. Reactions (50 μL) were initiated by adding 5 μL of extract and were stopped with 15 μL of 12 N hydrochloric acid. The reaction mixtures were dried at 55°C, and the solids were suspended in 30 μL of water and were counted in a 1450 liquid scintillation counter (PerkinElmer, Massachusetts, United States). Minus acetyl-CoA controls were included.

### Quantitative measurement of neutral lipids and confocal image observation

Neutral lipids were quantitatively measured using the procedure published by Chen *et al*. [55]. Chlorella cells grown under nutrient-limited condition were diluted to OD_750_ = 0.06, and 5 μL samples were introduced into the individual wells of a 96-microplate containing 3 μL of a 50 μg/mL Nile red solution (Sigma Aldrich, St. Louis, MO, United States). Then, 292 μL of an aqueous solution containing 25% dimethyl sulfoxide (Sigma Aldrich, St. Louis, MO, United States) was added. The 96-well plate was vortexed (120 rpm) and incubated at 40°C for 10 minutes. After the algal cells were stained, the fluorescence emission was recorded using a Varian spectrophotometer (Thermo Fisher Scientific, Rockford, IL, USA) equipped with a 96-well plate reader. Excitation and emission wavelengths of 530 nm and 575 nm, respectively, were selected. Eight replicates of each treatment were analyzed. Images were acquired using a Zeiss Cell Observer SD (Carl Zeiss Microscopy GmbH, Jena, Germany).

### Statistical analysis

All the experimental data were compared statistically through one-way analysis of variance (ANOVA) using the software Statistical Product and Service Solutions (SPSS) v17.0 followed by Student’s t-test to determine the significant difference among the treatment means.

### Accession number

The RNA-Seq data can be found in the GEO library under the accession number [GSE:37473].

## Additional files

Additional file 1: Figure S1. Size distribution of the contigs by RNA-seq. Additional file 2: Figure S2. Cumulative distribution of the log2 fold change in expression for lipid- and fatty acid-associated genes. The X-axis shows the value of the log2 fold change genes. The Y-axis shows the percentage of genes with a log2 fold change value greater than the corresponding value of the x-axis. There are more upregulated genes than downregulated genes associated with lipid and fatty acid metabolism. Additional file 3: Figure S3. Characterization of transgenic *C. ellipsoidea* expressing *GmDof4* under autotrophic culture conditions. **(A)** Growth curves of transgenic *C. ellipsoidea* expressing *GmDof4* under autotrophic culture conditions for 16 days. **(B)** Growth rate of transgenic *GmDof4* strains compared with the control under autotrophic culture conditions. Additional file 4: Figure S4. Determination of the *GmDof4* gene expression in different transgenic lines by quantitative RT-PCR. Additional file 5: Table S1. Lipid productivity of *C. ellipsoidea* under autotrophic culture conditions. Additional file 6: Table S2. Primers used in vector construction and transformant confirmation. **Table S3.** Primers used in the quantitative real-time PCR confirmation of differential gene expression.

## Abbreviations

ACCase: Acetyl-coenzyme A carboxylase
BWA: Burrows-Wheeler alignment tool
dCTP: Deoxycytidine 5′-triphosphate
Dof: DNA binding with one finger
FAS: Fatty acid synthase
GO: Gene ontology
GEO: Gene Expression Omnibus
Nr: Non-redundant
qRT-PCR: Quantitative real-time polymerase chain reaction
RT-PCR: Reverse transcription polymerase chain reaction
SE: Selenite Enrichment
TFs: Transcription factors
