# Supplementary figures and images for: Overexpression of the soybean transcription factor GmDof4 significantly enhances the lipid content of Chlorella ellipsoidea
Source: Biotechnol Biofuels. 2014 Sep 4;7:128. doi: 10.1186/s13068-014-0128-4 (PMC4159510; doi:10.1186/s13068-014-0128-4)

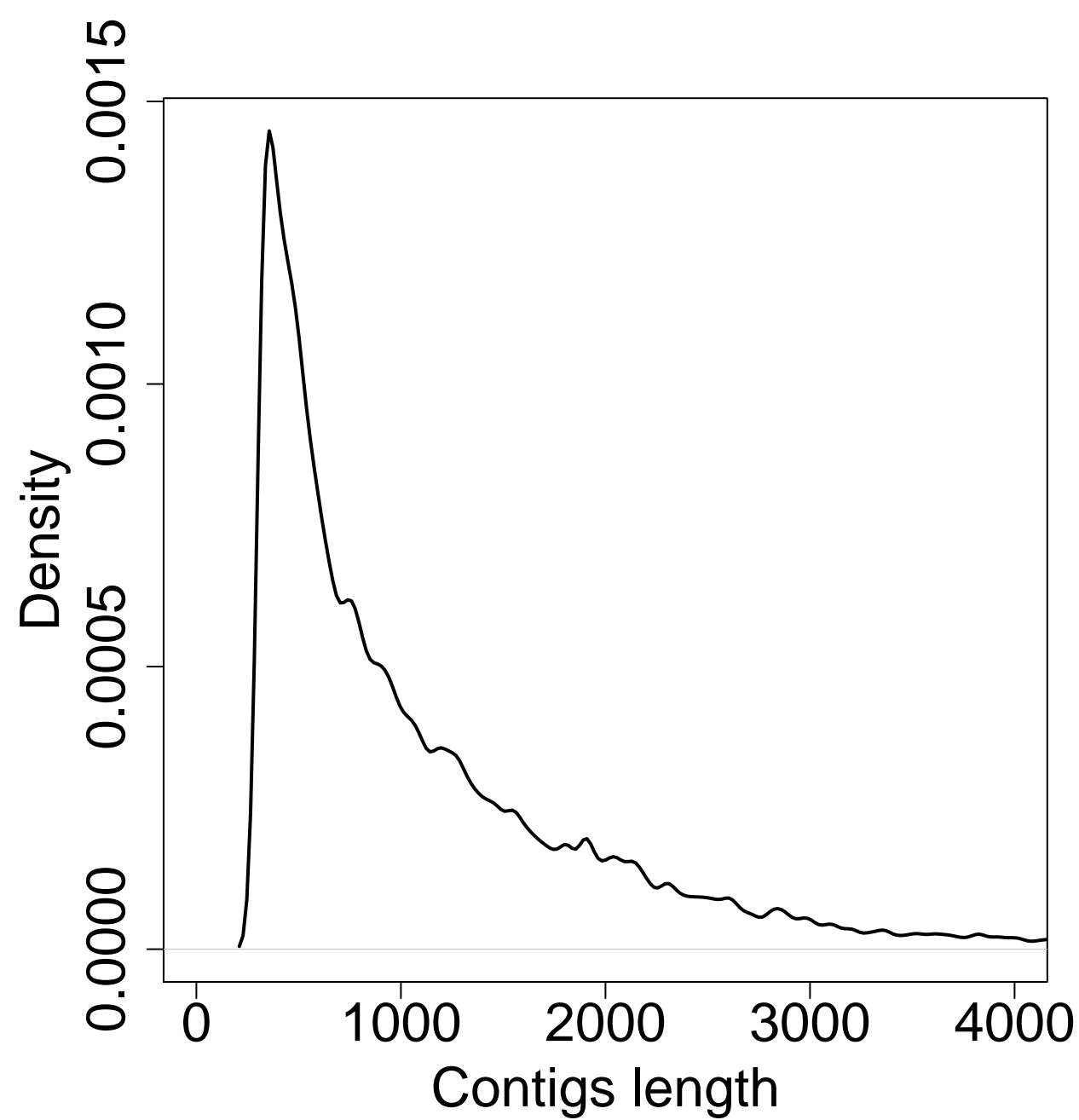

Supplement: Additional file 1: Figure S1. — Size distribution of the contigs by RNA-seq. [file 13068_2014_128_MOESM1_ESM.pdf]

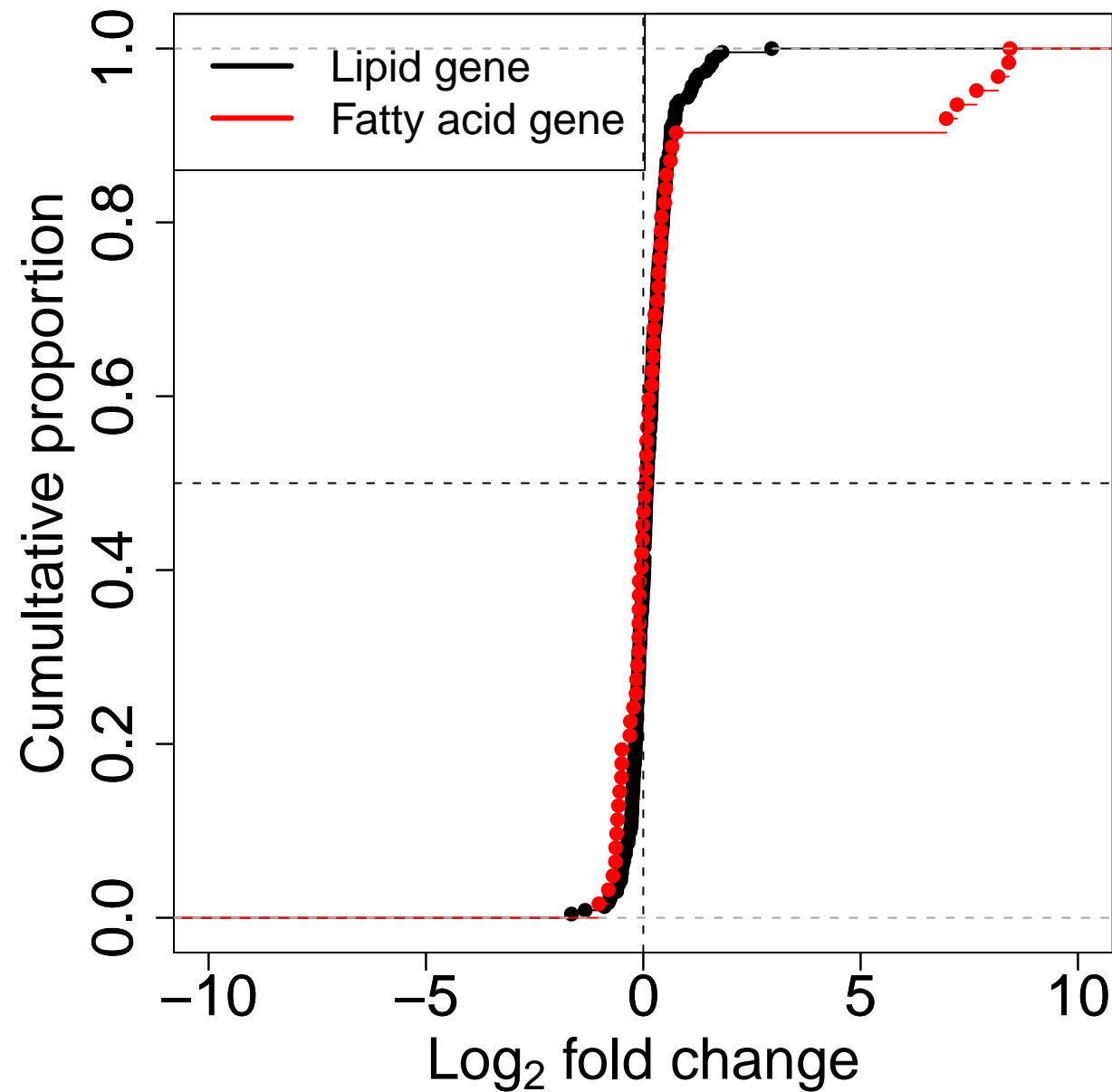

Supplement: Additional file 2: Figure S2. — Cumulative distribution of the log2 fold change in expression for lipid- and fatty acid-associated genes. The X-axis shows the value of the log2 fold change genes. The Y-axis shows the percentage of genes with a log2 fold change value greater than the corresponding value of the x-axis. There are more upregulated genes than downregulated genes associated with lipid and fatty acid metabolism. [file 13068_2014_128_MOESM2_ESM.pdf]

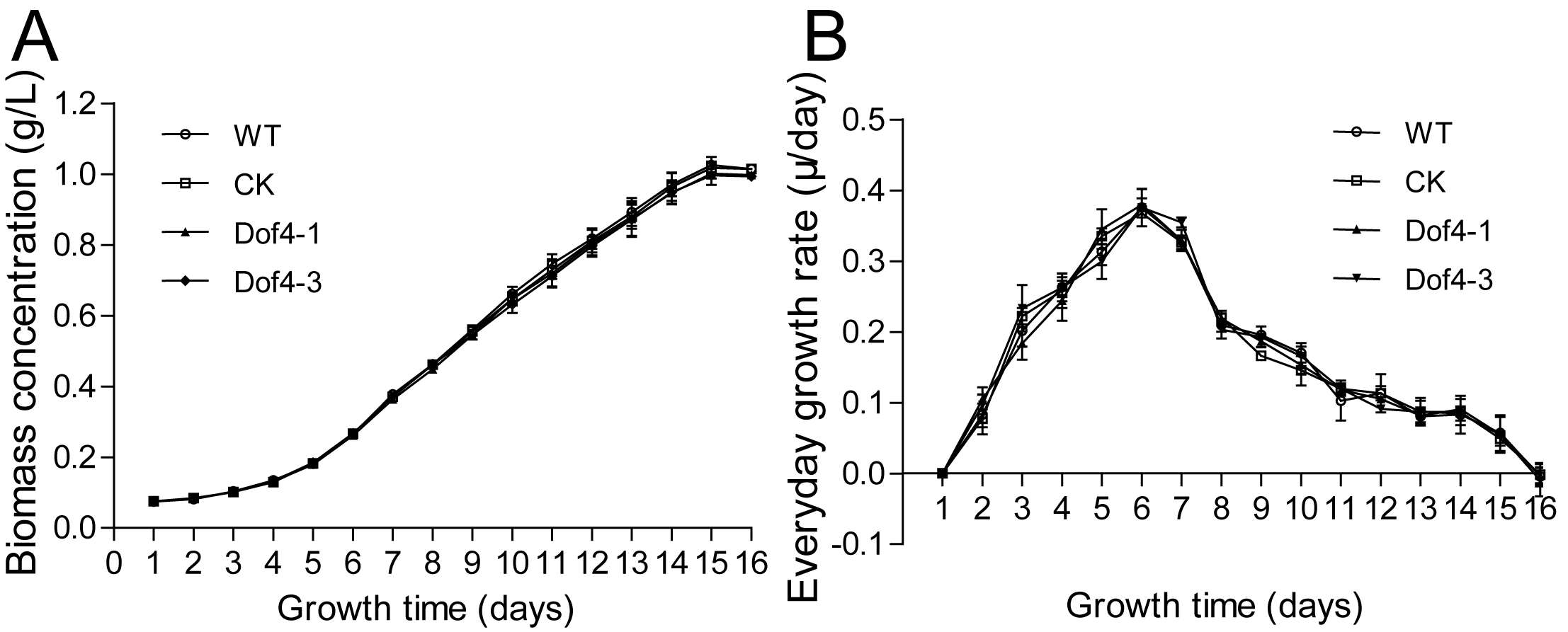

Supplement: Additional file 3: Figure S3. — Characterization of transgenic C. ellipsoidea expressing GmDof4 under autotrophic culture conditions. (A) Growth curves of transgenic C. ellipsoidea expressing GmDof4 under autotrophic culture conditions for 16 days. (B) Growth rate of transgenic GmDof4 strains compared with the control under autotrophic culture conditions. [file 13068_2014_128_MOESM3_ESM.png]

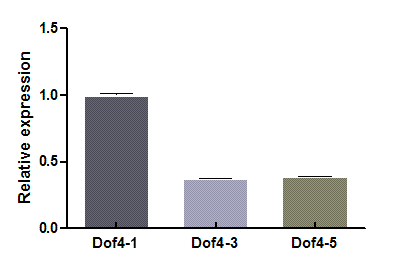

Supplement: Additional file 4: Figure S4. — Determination of the GmDof4 gene expression in different transgenic lines by quantitative RT-PCR. [file 13068_2014_128_MOESM4_ESM.png]
