# Supplementary material for: Overexpression of the soybean transcription factor GmDof4 significantly enhances the lipid content of Chlorella ellipsoidea
Source: Biotechnol Biofuels. 2014 Sep 4;7:128. doi: 10.1186/s13068-014-0128-4 (PMC4159510; doi:10.1186/s13068-014-0128-4)
Supplement: Additional file 5: Table S1. — Lipid productivity of C. ellipsoidea under autotrophic culture conditions. [file 13068_2014_128_MOESM5_ESM.pdf]

Table S1 Lipid productivity of *C. ellipsoidea* under autotrophic culture conditions. The data represent means ± SD of three replicate experiments and was analyzed by Student's *t* test (n=3). *, *P*<0.05.

	WT	CK	Dof4-1	Dof4-3
Lipid productivity (mg/L/d)	11.03±0.13	10.90±0.14	15.60±0.23*	15.70±0.27*
