# Supplementary material for: Overexpression of the soybean transcription factor GmDof4 significantly enhances the lipid content of Chlorella ellipsoidea
Source: Biotechnol Biofuels. 2014 Sep 4;7:128. doi: 10.1186/s13068-014-0128-4 (PMC4159510; doi:10.1186/s13068-014-0128-4)
Supplement: Additional file 6: Table S2. — Primers used in vector construction and transformant confirmation. Table S3. Primers used in the quantitative real-time PCR confirmation of differential gene expression. [file 13068_2014_128_MOESM6_ESM.pdf]

Table S2 Primers used in vector construction and transformants confirmation

Experiment	Name of primers	Sequence (5' to 3')
Amplifying *GmDof4* cDNA	P1	GCGactagtATGCAGCAAATACACT
	P2	GCGgcggccgcTCAGGGAAGATGAAAG
Obtaining *nos* terminator	P3	ATAAGAATgcggccgcTCGAATTTCCCCGATCGTTCAAAC
	P4	CgagctcGCCCGATCTAGTAACATAGATGA
Obtaining *ubi* promoter	P5	GCGaagcttGCATGCCTGCAGTGCAGCGTGACCCG
	P6	GCGggatccTCTAGAGTCGACCTGCAGAAGTAACACCAA
Obtaining *GmDof4* probe for Southern blot	P7	GCCGCTATTGGACTAAAGG
	P8	CGACAAATCAACGGTGTG
RT-PCR of *GmDof4*	P9	TGCGACTCTCTCAACACCA
	P10	GAAGAAGAATTGTCGTGG
RT-PCR of *npt* II	P11	GGAGAGGCTATTCGGCTATG
	P12	GCCAACGCTATGTCCTGATA

Sequences in lower-case letters indicate enzyme restriction sites.

Table S3 Primers used in Real-time PCR confirmation of differential gene expression

Gene ID	Forward primer (5’ to 3’)	Reverse primer (5’ to 3’)
67502	TGGGAACCAGACTTGCCCT	GACGCTGCTCAACATCCCA
56171	AGCAGGCAGGTCAGGCTAT	CATGGGCGACAACTATGTGGA
80365	GAGGGACTCACCATCACTATG	TGACTCACACCATTCACCC
101511	GAGGCGGAAGAGCTGGTTATG	TTTGAGGGTGAGCAGCCAG
71421	AGATCGGACTGGTGGACTCT	GCACGCTGATGGAGGTGTA
91597	CTCAGCACCCTCAGCTCATT	GTACAGCTAGGTCTCGCAAGG
86271	AGCCACAACAAGCCAGGAA	AAGTGGCTGGTGGACCTGAT
83574	TCTGGGAGTTTGTGGACAC	AGGTGATGCTCTTCTTCAGC
69772	ATGAGCCGCAGGTTCTCTTC	GACTTGGAAGACCCACAGCA
85953	CTAGATTGCCGTAGCCGAGG	CCAAAGGCGAGGCTGTTCTA
76019	CCTGTTGCCTCAATCAGCA	ATGTGTCTGGGCGTGTGAA
91381	ATCCTAGCAGCGTGACAACA	ACACCAGCGTACTGATGCTC
85042	AGTTGATGGCAAAGGAGCG	AGGAAACCCTGACCCTGACT
78075	CCATAGAATCTGGCGATGAAC	TACAGCACAACTGAGGCACC
88610	TGGTATGACATCTACAGCATCG	AGTTTCTCGTCTGCCATCG
88508	TTCTTCAAGCTGGGAATGCTCT	TGCAGAACAATGGCACCAATC
86121	AACCTGACTATCCTGGCACC	GCATTCCTTGTTTGCTCTCA
59360	TCGAGTTCCAGGTGCTCTGT	TCTGGTTGCGTCTCTGGATG
76421	TCCATGTTGAACGCACCCA	TTGAGCTGGCAGAGAGCATT
82144	TCCACTTTCAGGATTGTTGC	CGAATGATTATTGCCCAGG
83046	GCGAGATTTGGATGACCA	CAATGGGTTACGAGGTTCC
59485	AGGTCAAGCCCGACAGTAAC	GGTGCTCCTCAAATAGCGA
GmDof4	ATTCGGATCGTTGGATTGGCA	AGAGAAGAAGAATTGTCGTGGTCA
18S rRNA	CTTGTAAACCGCGTCGTGATG	GACGTAATCAACGCGAGCTGAT
